# The function of cancer-shed gangliosides in macrophage phenotype: involvement with angiogenesis

**DOI:** 10.18632/oncotarget.13878

**Published:** 2016-12-10

**Authors:** Tae-Wook Chung, Hee-Jung Choi, Mi-Ju Park, Hee-Jin Choi, Syng-Ook Lee, Keuk-Jun Kim, Cheorl-Ho Kim, Changwan Hong, Kyun-Ha Kim, Myungsoo Joo, Ki-Tae Ha

**Affiliations:** ^1^ Korean Medical Research Center for Healthy Aging and Yangsan, Gyeongsangnam-do, Republic of Korea; ^2^ School of Korean Medicine, Pusan National University, Yangsan, Gyeongsangnam-do, Republic of Korea; ^3^ Department of Food Science and Technology, Keimyung University, Daegu, Republic of Korea; ^4^ Department of Clinical Pathology, TaeKyeung University, Gyeongsan, Gyeongsangbuk-do, Republic of Korea; ^5^ Department of Biological Science, Sungkyunkwan University, Suwon, Kyunggi-do, Republic of Korea; ^6^ Department of Anatomy, School of Medicine, Pusan National University, Yangsan, Gyeongsangnam-do, Republic of Korea

**Keywords:** ganglioside, tumor-associated macrophage, macrophage mannose receptor, monocyte chemoattractant protein-1, angiogenesis

## Abstract

Tumor-derived gangliosides in the tumor microenvironment are involved in the malignant progression of cancer. However, the molecular mechanisms underlying the effects of gangliosides shed from tumors on macrophage phenotype remain unknown. Here, we showed that ganglioside GM1 highly induced the activity and expression of arginase-1 (Arg-1), a major M2 macrophage marker, compared to various gangliosides in bone marrow-derived macrophages (BMDM), peritoneal macrophages and Raw264.7 macrophage cells. We found that GM1 bound to macrophage mannose receptor (MMR/CD206) and common gamma chain (γc). In addition, GM1 increased Arg-1 expression through CD206 and γc-mediated activation of Janus kinase 3 (JAK3) and signal transducer and activator of transcription- 6 (STAT-6). Interestingly, GM1-stimulated macrophages secreted monocyte chemoattractant protein-1 (MCP-1/CCL2) through a CD206/γc/STAT6-mediated signaling pathway and induced angiogenesis. Moreover, the angiogenic effect of GM1-treated macrophages was diminished by RS102895, an MCP-1 receptor (CCR2) antagonist. From these results we suggest that tumor-shed ganglioside is a secretory factor regulating the phenotype of macrophages and consequently enhancing angiogenesis.

## INTRODUCTION

Tumor-associated macrophages (TAMs) play key roles in the pathogenesis of solid tumors by enhancing tumor growth, invasion, metastasis, immune suppression, and angiogenesis [1]. The functional phenotypes of TAMs are regulated by soluble factors released from tumor cells, lymphocytes, and stromal cells in the tumor microenvironment [2]. Tumor derived molecules, such as IL-4, IL-10, and transforming growth factor-β1 (TGF-β1), have been proposed as major factors inducing M2-polariziation of TAMs [3, 4]. However, the various factors leading to M2 phenotype expression of TAMs are still not fully elucidated.

Gangliosides are sialic acid-containing complex glycosphingolipids mainly located on the outer leaflet of the plasma membrane. However, most tumor cells synthesize and shed large numbers of gangliosides into the extracellular environment, as free molecules, micelles, protein-bound complexes or membrane fragments [5, 6]. Tumor-shed gangliosides have pleiotropic effects, inducing regulation of tumor growth [7], angiogenesis [8], and immune modulation [9]. However, there is no direct evidence that reveals the function of tumor-shed gangliosides on the plasticity of macrophages in the tumor microenvironment.

In this study, GM1 was selected for the mechanism studies because it was the most active on the expression of Arg-1, a major M2 phenotype marker of macrophage in all of BMDM, peritoneal macrophage and macrophage Raw264.7 cells. Thus, we report, for the first time, the finding that ganglioside GM1 interacts with CD206 and γc to activate JAK3/STAT6 signaling pathway. Furthermore, GM1 has an effect on macrophage phenotype by not only regulating the expression of Arg-1 but by inducing the secretion of MCP-1 through CD206-mediated activation of STAT6, which enhances *in vitro* and *in vivo* angiogenesis, resulting in activation of endothelial cells.

## RESULTS

### Cancer-shed GM1 increases Arg-1 expression of macrophages

**Figure 1:**
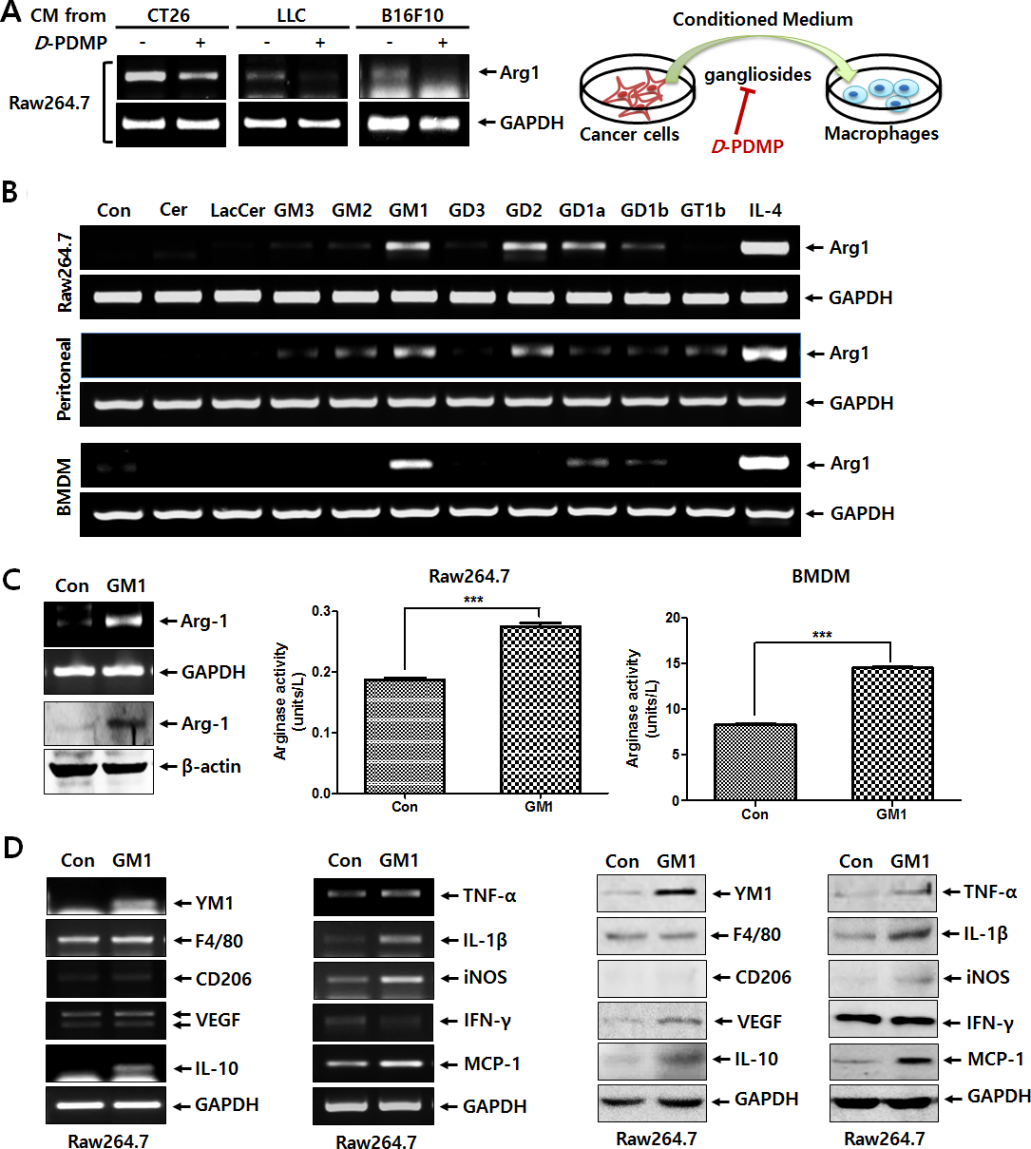
Tumor-shed gangliosides increase the expression of Arg-1 in macrophages (**A**) The CT26, LLC, and B16F10 cells (5 × 10^5^) suspended in 2 ml of culture medium were seeded on 6-well plates, treated with or without *D*-PDMP (10 μM), and incubated at 37°C in 5% CO_2_ atmosphere. At 24 h after incubation, one milliliter of conditioned medium harvested from cancer cells was added to 6-well plates seeded with Raw264.7 macrophage cells (1 × 10^6^ cells), which were incubated at 37°C in 5% CO_2_ atmosphere for 24 h. Arg-1 expression of was determined by RT-PCR. (**B**) Ceramide or diverse glycosphingolipids, including LacCer, GM3, GM2, GM1, GD3, GD2, GD1a, GD1b and GT1b (10 μM, respectively), were incubated with Raw264.7 cells, peritoneal macrophages and BMDMs for 24 h. Arg-1 expression was analyzed by RT-PCR. IL-4 (20 ng/ml) was used as a positive control. (**C**) Raw264.7 cells were treated with GM1 (10 μM) for 24 h. Arg-1 expression was examined by RT-PCR and western blot analysis. Arginase enzyme activity was measured using cell lysates obtained from Raw264.7 cells (1 × 10^6^ cells) and BMDMs (3 × 10^6^ cells) treated with GM1 (10 μM). (**D**) Raw264.7 cells were treated with GM1 (10 μM) for 24 h, and expression of typical markers of M1 or M2 macrophage phenotypes was examined by RT-PCR and Western blot analysis.

We hypothesized that tumor-shed gangliosides, which have diverse cellular functions in the tumor microenvironment [7, 8, 10], may modulate macrophage phenotype to the advantage of cancer growth. As shown in Figure 1A, we confirmed that the expression of Arg-1, a key marker of macrophage M2 polarization, is induced in Raw264.7 macrophage cells treated with conditioned culture media obtained from 3 different tumor cell types, including CT26 colon carcinoma cells, Lewis lung carcinoma LLC cells, and B16-F10 melanoma cells. In contrast, use of conditioned media from cancer cells treated with *D*-PDMP, an inhibitor of glucosylceramide synthase and lactosylceramide synthase, to inhibit ganglioside synthesis [11], resulted in the reduction of Arg-1 expression (Figure 1A). These results suggest that gangliosides might be involved in the induction of Arg-1 expression in tumor culture media-activated macrophages.

To identify the ganglioside responsible for the induction of Arg-1 expression, three types of macrophages, including Raw264.7 cells, peritoneal macrophages, and BMDMs were treated with ceramide and lactosylceramide as precursors of gangliosides and diverse gangliosides. As shown in Figure 1B, GM1 caused the highest induction of Arg-1 expression in all 3 macrophage lineages. The effect of GM1 on the upregulation of Arg-1 expression was also confirmed by western blot analysis and arginase activity assay in Raw264.7 and BMDM cells (Figure 1C). Furthermore, as shown in Figure 1D, GM1 induced the expression of several M2 markers, such as YM1, VEGF and IL-10 and M1 markers, including TNF-α, IL-1β, iNOS, and MCP-1. The expression of F4/80 and CD206, typical M2 markers, was not increased significantly by GM1. The expression of IFN-γ, an M1 marker, was slightly decreased. These results indicate that GM1-stimulated macrophages do not show typical M2 polarization, although the expression of Arg-1, as a key marker of M2 phenotype was elevated by GM1 treatment.

### GM1 enhances Arg-1 expression by activating CD206/γc complex and JAK3/STAT6 signaling pathway

**Figure 2:**
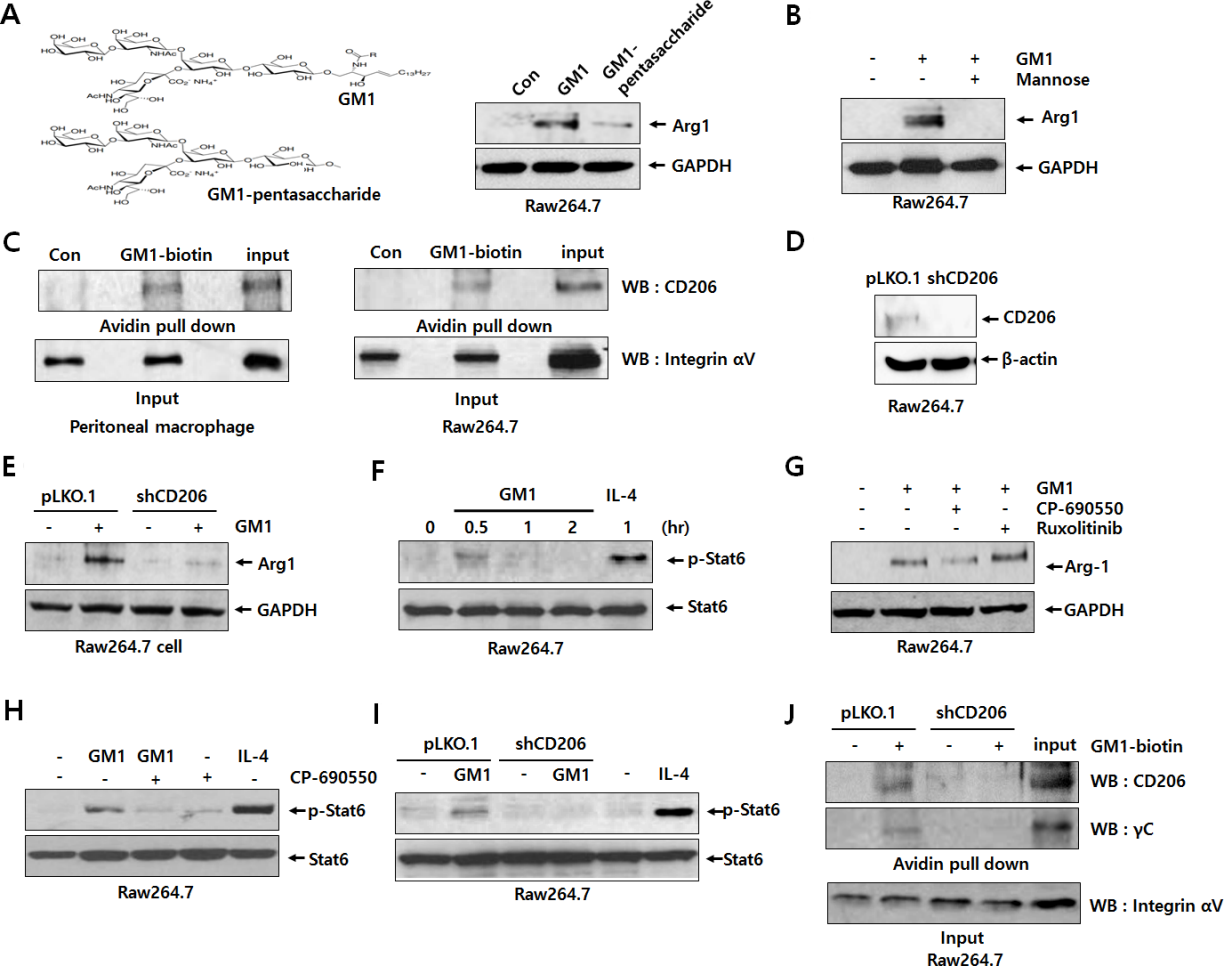
GM1 activates Arg-1 expression through interaction with CD206/γc and downstream signaling of JAK3/STAT6 (**A**) Schematic representation of GM1 and GM1-pentasacchride effects on Arg-1 expression. Raw264.7 cells were treated with 10 μM of GM1, and GM1-pentasaccharide. Arg-1 expression was determined by western blot analysis. (**B**) The inhibitory effect of mannose on GM1-stimulated Arg-1 expression was assessed by western blot analysis. Raw264.7 cells were treated with mannose (10 μM) for 1 h, and then GM1 was added for 24 h. (**C**) To confirm binding between GM1 and CD206, cell lysates from peritoneal macrophages and Raw264.7 cells were subjected to pull-down reactions using GM1-biotin and incubated with avidin-beads. Precipitated proteins were resolved and applied to western blot analysis. (**D**) Expression of CD206 by Raw264.7 cells transfected with control (pLKO.1) or shRNA (shCD206) vectors was confirmed by western blot analysis. (**E**) Expression of CD206 and Arg-1 by GM1-treated Raw264.7 cells transfected with control or shRNA vector was analyzed by western blot analysis. (**F**) Time-course effect of GM1 on the phosphorylation of STAT6 in Raw264.7 cells was determined by western blot analysis. IL-4 was used as a positive control. (**G**) Effects of JAK inhibitors CP-690550 (for JAK3; 100 nM) and Ruxolitinib (for JAK1/2; 100 nM) on the GM1-stimulated Arg-1 expression were evaluated by western blot analysis. Raw264.7 cells were incubated with CP-690550 or Ruxolitinib for 1 h, then treated with GM1 (10 μM), and cultured for 24 h. (**H**) Effect of GM1 and CP-690550 on the phosphorylation of STAT6 was examined by western blot analysis. After Raw264.7 cells were treated with CP-690550 for 1 h, and the cells were incubated with GM1 for 30 min. (**I**) Raw264.7 cells transfected with control (pLKO.1) or shRNA (shCD206) vectors were treated with GM1 (10 μM) for 30 min. Phosphorylation of STAT6 was confirmed by western blot analysis. (**J**) To check interaction of CD206 and γC with GM1, cell lysates from Raw264.7 cells transfected with control (pLKO.1) or shRNA (shCD206) vectors were applied to pull-down reactions using GM1-biotin. After incubation with avidin-beads, precipitated proteins were resuspended and subjected to western blot analysis.

To understand the interaction between cancer-shed GM1 and molecules on the macrophage plasma membrane, we treated macrophages with a GM1 carbohydrate moiety harboring no lipid tail (ceramide-removed) to prevent exogenous GM1 from incorporating into the plasma membrane. Interestingly, similar to GM1, GM1-pentasaccharide also increased the expression of Arg-1 (Figure 2A). Thus, we assumed that the GM1 carbohydrate moiety interacted with surface proteins, such as lectins, recognizing specific carbohydrates on the plasma membrane. To elucidate which lectin is involved in GM1-stimulated Arg-1 expression, macrophages were treated with several monosaccharides, including *D*-mannose, *D*-galactose, *D*-glucose, and *L*-fucose, reported to be lectin inhibitors [12]. Among them, mannose specifically blocked GM1-activated Arg-1 expression (Figures 2B and S1). Since mannose is known as a specific inhibitor for the macrophage mannose receptor (CD206) [13], this result suggests that CD206 may be responsible for the GM1-induced expression of Arg-1. Thus, to examine whether CD206 can actually bind to carbohydrate residues on GM1, we performed a pull-down assay using GM1 analogue labeled with biotin for its amino residue on the sphingosine moiety [14]. Interestingly, CD206 expressed on the membrane of peritoneal macrophages and Raw264.7 cells potentially interacts with GM1 (Figure 2C).

To elucidate the role of CD206 in GM1-induced Arg-1 expression, we created CD206 knock-down cells using shRNA against CD206. As shown in Figure 2D, the expression of CD206 was diminished in the cells transfected with shCD206. Using these cells, we confirmed that GM1-enhanced expression of Arg-1 was markedly decreased by knock-down of CD206 (Figure 2E). These results suggest that the function of CD206 is crucial to GM1-induced Arg-1 expression.

It has been reported that Arg-1 expression is mainly increased by JAK/STAT activation [15, 16]. Thus, we also investigated the phosphorylation of STATs in GM1-treated macrophage cells using western blot analysis. As shown in Figures 2F and S3, STAT6 was activated at 30 min after GM1 treatment, but other STATs, including STAT1, STAT3, and STAT5, were not. To figure out which type of JAK is responsible for GM1-induced phosphorylation of STAT6, we used specific signal inhibitors against JAKs, including the JAK3 antagonist CP-690550 and JAK1/2 inhibitor Ruxolitinib [17]. The results showed that GM1-induced Arg-1 expression and STAT6 phosphorylation were reduced by treatment with CP-690550 (Figure 2G and 2H). In addition, GM1-induced activation of STAT6 was markedly diminished by knock-down of CD206 expression (Figure 2I). Thus, we supposed that GM1-induced signal activation is mediated by the CD206/JAK3/STAT6 pathway for Arg-1 expression.

Unlike other cytokine receptor-JAK associations, it has been reported that JAK3 selectively interacts with γc [17, 18]. Thus, we hypothesized that cancer-shed GM1 may directly interact with γc on the plasma membrane of macrophages, or form complexes with CD206 and γc. To test this hypothesis, we confirmed the interaction of GM1 and γc by pull-down assay using GM1-biotin as a probe. As shown in Figure 2J, for the first time, we found that biotin-labeled GM1 could bind to γc as well as CD206.

### GM1-stimulated macrophages activate angiogenesis via MCP-1/CCR2 interaction

**Figure 3:**
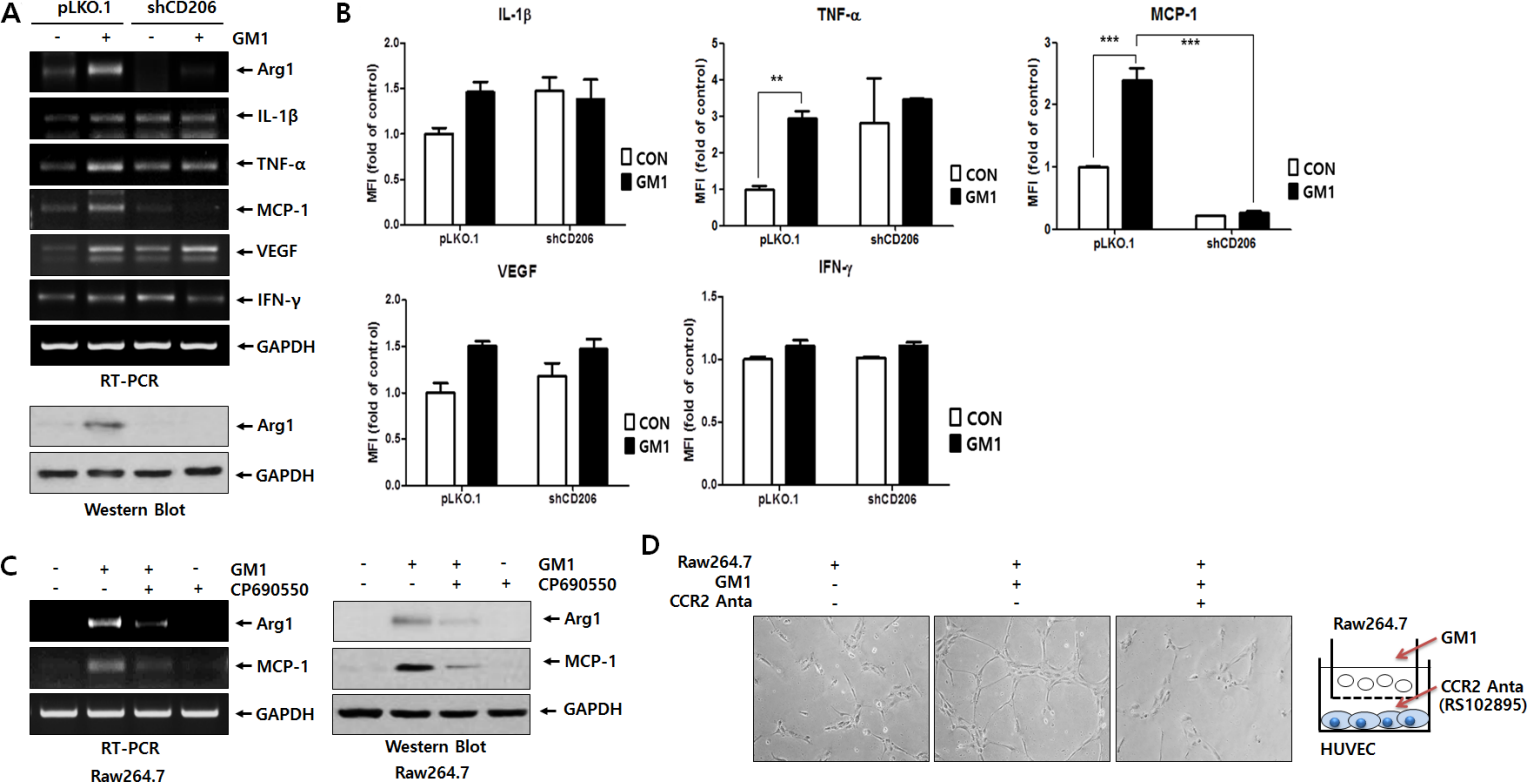
GM1 enhances HUVEC tube formation via secretion of MCP-1 from Raw264.7 cells GM1 (10 μM) was added to Raw264.7 cells transfected with control (pLKO.1) or shRNA (shCD206) vectors for 24 h. (**A**) Effect of GM1 on the expression of cytokines and growth factors IL-1β, TNF-α, MCP-1, VEGF, and IFN-γ in control or CD206 knock-down Raw264.7 cells was evaluated by RT-PCR. Arg-1 expressions was determined by RT-PCR and Western blot analysis. (**B**) Secretion of IL-1β, TNF-α, VEGF, IFN-γ and MCP-1 were analyzed by Luminex multiplexing system. (**C**) Raw264.7 cells were treated with CP-690550 (10 μM) for 1 h before GM1-stimulation, and the cells were incubated for 24 h. Expression of Arg-1 and MCP-1 was estimated by RT-PCR and Western blot analysis. (**D**) Raw264.7 cells and HUVECs were co-cultured in a boyden chamber system. Raw264.7 cells in the upper chamber were activated with GM1 and CCR2 antagonist (RS102895; 10 μM) was added to HUVECs cultured in the Matrigel-coated lower chamber. After 12 h incubation, formation of HUVEC tubular structures was observed by microscopy and representative pictures are shown.

Figure S2 and Figure 1D show that the production of several cytokines and growth factors, including IL-1β, TNF-α, MCP-1, and VEGF, is increased by GM1 treatment. Thus, we investigated whether the expression of these cytokines and growth factors is linked to the CD206 pathway by comparing their expression in pLKO.1 and shCD206 macrophages. As evidenced by RT-PCR and Luminex multiplexing assay, the expression of MCP-1 was clearly induced by GM1 in a CD206-responsible manner (Figure 3A and 3B). On the other hand, although the expression levels of IL-1β, TNF-α and VEGF mRNA were increased by GM1, their expression did not seem to be mediated by CD206 (Figure 3A and 3B). Furthermore, the GM1-stimulated expression of Arg-1 and MCP-1 was also reduced by treatment with the JAK3 antagonist, CP690550 (Figure 3C). Collectively, these results show that GM1-enhanced expression of Arg-1 and MCP-1 is induced in a CD206- and JAK3- dependent manner, as shown in Figure 2.

**Figure 4:**
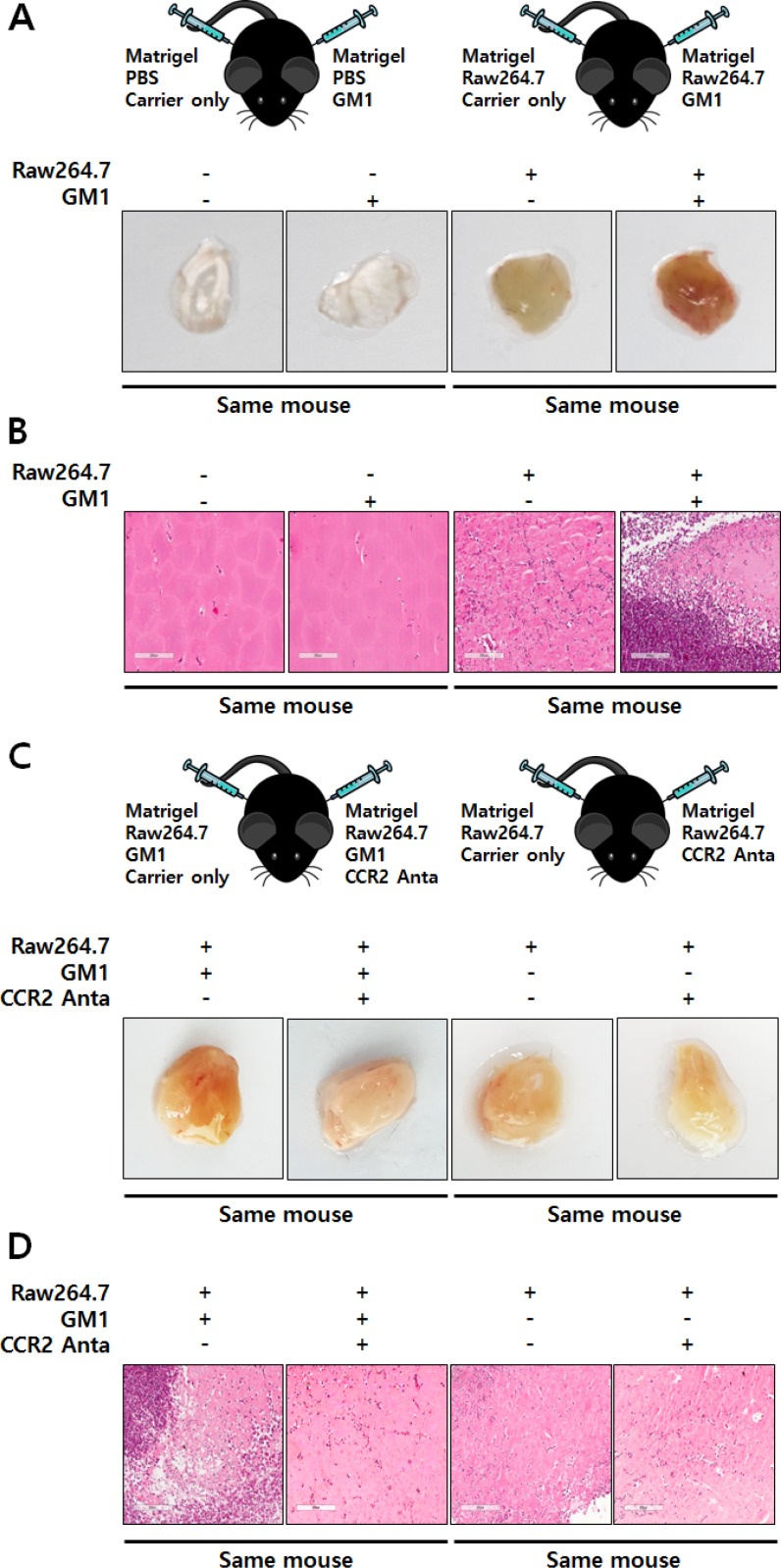
GM1-stimulated macrophages induce *in vivo* angiogenesis through functional activation of CCR2 (**A**) Matrigels containing Raw264.7 cells and/or GM1 were subcutaneously injected into the abdomen of C57BL/6 mice. After 7 days, the mice were sacrificed, the matrigels were collected, and images were recorded. Representative images are demonstrated. (**B**) Matrigels were fixed, sectioned, and stained with H&E. Representative are shown. (**C**) Matrigels harboring Raw264.7 cells supplemented with GM1 and/or an antagonist against CCR2 were injected subcutaneously into the abdomen of mice. After 7 days, the mice were sacrificed and matrigels were collected. Gross images were recorded and representative images are presented. (**D**) Matrigels were fixed, sectioned, and stained with H&E; Representative images are shown.

Although the best-known role of MCP-1 is modulation of the inflammatory response by inducing monocyte recruitment, it also directly affects angiogenesis [19–21]. Thus, to examine the angiogenic effect of GM1-activated macrophages, we co-cultured HUVECs and Raw264.7 cells. The results showed that GM1-stimulated macrophages induced tubular morphological formation of HUVECs. However, HUVEC tube formation was reduced after treatment with RS102895, an antagonist for CCR2, the MCP-1 receptor (Figure 3D). To confirm the *in vivo* angiogenic effect of GM1-treated macrophages, we performed a Matrigel plug assay mixed with macrophages, as described in previous study [22]. The results showed that GM1-stimulated macrophages enhanced the infiltration of vessel cells, evidenced by gross and microscopic observations. However, GM1 itself did not affect *in vivo* angiogenesis (Figure 4A and 4B) or HUVEC tube formation (data not shown). In addition, angiogenesis induced by co-treatment of GM1 and macrophages was reduced by the addition of the CCR2 antagonist (Figure 4C and 4D). These results clearly suggest that *in vitro* and *in vivo* angiogenesis induced by GM1-stimulated macrophages is mediated by interactions between MCP-1 and CCR2.

## DISCUSSION

This study showed that tumor-shed ganglioside stimulated macrophages in the microenvironment network. Subsequently, activated macrophage secreted MCP-1 to activate endothelial cells for angiogenesis in the cancer microenvironment to benefit cancer growth. Angiogenesis is a crucial process for the growth and progression of human cancers. Tumor angiogenesis is induced not only by the interaction between cancer cells and endothelial cells, but by infiltrated immune cells which have a key role in driving the formation of new blood vessels [23]. Among these immune cells, macrophages are the dominant cell type in quantity and in function [4]. Macrophages infiltrating in the tumor region are mainly derived from circulating monocytes and recruited at cancer sites by chemotactic factors, such as MCP-1 and regulated on activation, normal T cell expressed and secreted (RANTES) [24, 25]. Of the two major macrophage phenotypes (M1 and M2), the M2 phenotype of TAMs in the tumor region are basically regulated by a subset of tumor-secreted factors, such as M-CSF, IL-4, IL-13 and IL-10 [3, 14]. TAMs instigate their tumor-promoting action through suppression of immune surveillance and enhancement of neovascularization [14]. Angiogenic TAMs can drive tumor angiogenesis by producing various factors, such as VEGFs, FGFs, endothelin, IL-17, IL-23, TGF-β, and chemokines [8]. In a previous study, Manfredi *et al*., reported that ependymoblastoma tumor cells overexpressing *N*-acetylgalactosaminyl transferase (GNT), an important enzyme for the synthesis of complex gangliosides, induces the production of complex gangliosides GM2, GM1, and GD1a, resulting in increased tumor angiogenesis [26]. It has been reported that tumor-shed gangliosides are accelerating factors for angiogenesis [8, 26, 27], whereas anti-angiogenic or angiostatic effects of the simplest ganglioside GM3 were also reported [28, 29]. Although tumor angiogenesis was significantly reduced in tumor regions of mice injected with ganglioside-depleted tumor cells by double-knock out of *Siat9* and *Galgt1*, compared to those of mice injected with tumor cells, the production of VEGF and other angiogenic factors, such as cytokines and growth factors, in ganglioside-depleted tumor cells was not changed, compared to tumor cells [8]. Complex ganglioside synthesis enhanced *VEGF* gene expression in tumors in an *in vivo* mouse model bearing GNT-overexpressed tumor cells but not in *in vitro* GNT-overexpressed tumor cells. Furthermore, in Matrigel plugs containing EPEN-GNT tumor cells, angiogenesis was increased [26]. The Matrigel angiogenesis model and tumor injected mouse model are highly dependent upon host stromal cell infiltration and activation, including monocytes, macrophages, and endothelial precursors [26]. Ladisch group has been reported that tumor-shed gangliosides control the number and function of tumor-infiltrating myeloid-derived suppressor cells (MDSCs) [30] and tumor angiogenesis *in vivo* [8] using an *in vivo* model of genetic ganglioside depletion in tumor cells [31]. These results suggest that tumor-shed gangliosides directly activate endothelial cells, or may stimulate host cells to angiogenesis. Thus, we also assumed that tumor-shed ganglioside is one of the factors playing an important role in angiogenesis through regulation of macrophage phenotype in the tumor microenvironment. Among the multiple stromal cell types composing the tumor microenvironment, macrophages are the most abundant and are major regulators for fostering tumor progression [2]. Tumor-infiltrating macrophages express large amounts of o-series gangliosides, such as asialo-GM1, GM1b, and GD1α, compared with peritoneal macrophages [32]. In addition, gangliosides can suppress macrophage M1-like functions, including inhibition of Fc receptor expression, pro-inflammatory cytokine production, and antigen presentation [33–35]. However, the function of tumor-shed gangliosides on macrophage phenotype selection for angiogenesis in the tumor microenvironment has not been reported. In our experiments, ganglioside GM1 significantly induced expression of Arg-1, a major marker of M2 macrophages (Figure 1). Furthermore, the expression of M1 makers was also induced by GM1 (Figure 1), and GM1 clearly induced angiogenesis through the regulation of macrophage phenotype, as shown in *in vitro* and *in vivo* experimental models (Figures 3D and 4). These results indicate that although GM1-stimulated macrophages do not show typical M2 polarization, GM1 increases the expression of Arg-1, a key marker of the M2 macrophage phenotype, which results in the induction of angiogenesis.

GM1 ganglioside, an a-series ganglioside containing a single sialic acid residue, is the most widely used marker for lipid rafts in the plasma membrane [36]. Like other glycoconjugates, GM1 shed from cells mediates its biological function via interaction with soluble or membrane-bound molecules outside the cell (*trans*-interaction), or via influence on the proteins within the same membrane (*cis*-interaction) [25, 37] after incorporation into the lipid bilayer of the plasma membrane [38]. Thus, we confirmed how cancer-shed GM1 interact with molecules on the macrophage plasma membrane using its carbohydrate moiety. As shown in Figure 2A, GM1 pentasaccharide increased the expression of Arg-1. This result suggests that carbohydrate moiety of GM1 may increase Arg-1 expression through *tans*-interaction with surface proteins on the plasma membrane of macrophages, at least in part. It is well known that C-type lectins, a family of specific carbohydrate-recognizing proteins, are the most abundant lectins expressed on macrophages, and function as fundamental mediators of diverse immune interactions [39, 40]. Thus, to identify the lectin associated with GM1- induced Arg-1 expression, we used several monosaccharides as lectin inhibitors. From the results shown in Figures 2B and S1, we guessed that GM1 might interact with the macrophage mannose receptor, CD206 to induce Arg-1 expression based on mannose-specific inhibition. Data obtained using CD206 known down macrophage cells clearly showed that GM1 increased Arg-1 expression, resulting from binding of GM1 to CD206 (Figure 2C–2E).

CD206 can bind to terminal carbohydrate residues harboring sulfated or non-sulfated saccharides, such as mannose, fucose *N*-acetylglucosamine, *N*-acetylgalactosamine and galactose, via R-type or C-type carbohydrate-recognition domains (CRDs), respectively. The roles of CD206 are numerous and include clearance of endogenous molecules, promotion of antigen presentation, and modulation of cellular activation and trafficking [41]. Although the receptor does not have any motif to induce intracellular signals at its cytoplasmic tail, CD206 is essential for the production of both pro- and anti-inflammatory cytokines [42]. Furthermore, interactions of CD206 with toll-like receptor 2 (TLR2) or FcR were suggested as mechanisms for mediating activation of intracellular signaling [42, 43]. However, in this study, we wondered how to regulate CD206-mediated activation of intracellular signaling by GM1 for Arg-1 expression. It is well known that M1 and M2 polarization of macrophages is regulated by cellular signaling pathways stimulated with various signal activators, including cytokines [44]. Among the stimulators, IL-4, IL-13, or IL-10 results in the induction of M2-polarization of TAMs [3, 4]. It has been reported that Arg-1 is highly expressed by IL-4, which is considered a hallmark of M2 macrophages [45, 46]. Furthermore, the phosphorylation of STAT6 induced by IL-4 increases Arg-1 expression [46]. In immunoregulation and immune-mediated disease, STATs activation is closely associated with signal activation of JAKs [47]. In our data, GM1 markedly activated STAT-6, but not STAT-1, 3, or 5 in macrophage cells (Figures 2F and S3). In addition, the JAK3 inhibitor CP-690550 suppressed GM1-induced Arg-1 expression and STAT6 phosphorylation, as demonstrated by JAK1/2 and 3 signal inhibitors (Figure 2G and 2H). Moreover, in CD206-knock down macrophage cells, GM1 did not induce the phosphorylation of STAT6 (Figure 2I). These results demonstrate that CD206-mediated JAK3/STAT6 activation by GM1 is required for induction of Arg-1 expression.

Review articles by Rochman and O’Shea group showed that JAKs-STATs signaling pathways are critically involved in various immune responses among immune cells by the action of hormones, interferons (IFNs), growth factors, and interleukins [47, 48]. It is well known that cytokine receptor families are classified into immunoglobulin superfamily receptors, class I and II cytokine receptor families, the TNF receptor superfamily, and the chemokine receptor family [49]. Among these cytokine receptors, class I and II cytokine receptor families as receptors for ILs and IFNs mainly include JAKs-STATs downstream signaling pathways [49]. Furthermore, signal transducing chains of type I and II cytokine receptor families are often shared with common gamma chain, common beta chain, common alpha chain and gp130 receptors [47–50]. Review papers have shown that JAK3 is required for signaling of the type I receptors (IL-2, IL-4, IL-7, IL-9, IL-15, IL-21 receptors) that use the γc [48, 51]. In our previous data, treatment with the JAK3 inhibitor CP-690550 and CD206 knock-down in macrophage cells resulted in the inhibition of not only STAT6 activation but Arg-1 expression induced by GM1 in macrophage cells (Figure 2E–2I). Thus, we considered the association of CD206 with γc by GM1. To the best of our knowledge, this result is the first report on the interaction of ganglioside GM1 with γc in a CD206-dependent manner. To elucidate detailed conditions for the binding of GM1 to membrane receptors, including CD206 and γc, further extensive studies are need.

It is well known that MCP-1 is involved in monocyte and macrophage recruitment into various solid tumors [52]. In addition, tumors secrete MCP-1, resulting in TAM accumulation [52]. Salcedo *et al*. have reported that MCP-1 angiogenic effects were accompanied by monocyte-macrophage infiltration [21]. Moreover, a direct effect of MCP-1 on angiogenesis was consistent with the expression of the MCP-1 receptor (CCR2) on endothelial cells [21]. Interestingly, as evidenced by RT-PCR and Luminex multiplexing assay (Figure 3), MCP-1 expression was clearly increased by GM1 via the CD206/JAK3-mediated pathway in macrophage cells, similar to the regulation of Arg-1 expression by GM1 (Figure 2). Furthermore, MCP-1 released from macrophages stimulated by GM1 induced the activation of endothelial cells, which was inhibited by a CCR2 antagonist (Figures 3D and 4). Based on our data, CD206 seems to be a key molecule regulating cellular activation induced by GM1, of the angiogenic properties of macrophage.

**Figure 5:**
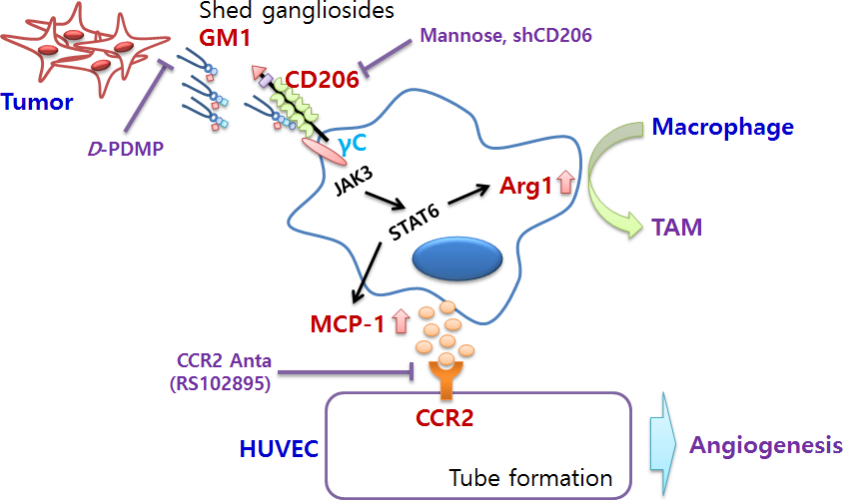
Schematic representation of pro-angiogenic effects of cancer-shed GM1 Tumor-shed gangliosides, especially GM1, regulates macrophage phenotype by activating CD206/γc complexes and the JAK3/STAT6 signaling pathway. Activated TAMs increase the expression of arginase-1, a typical marker of M2 phenotype, and production of MCP-1. Enhanced secretion of MCP-1 by GM1-stimulated macrophages induces *in vitro* and *in vivo* angiogenesis through ligation with MCP-1 receptor, CCR2, expressed in vascular endothelial cells.

In this study, we have demonstrated for the first time that tumor-shed gangliosides, especially monosialoganglioside GM1, greatly increased the expression of Arg-1, a prominent marker of M2 polarization in macrophages. The molecular signaling mechanism underlying GM1-induced expression of Arg-1 is linked to interaction with CD206 and γc, and subsequently the JAK3/STAT6 signaling pathway. In addition, GM1-stimulated macrophages produce high amounts of MCP-1 through CD206-mediated JAK3 activation and induce *in vitro* and *in vivo* angiogenesis. Moreover, the angiogenic effect of GM1-treated macrophages was abolished by a CCR2 antagonist (Figure 5). From these results we suggest that tumor-shed ganglioside induces angiogenesis by changing macrophage phenotype for tumor progression.

## MATERIALS AND METHODS

### Materials

Monosialoganglioside GM1 and *N*-Hexanoyl-biotin-monosialoganglioside GM1 (GM1-biotin) were purchased from Matreya LLC (State College, PA, USA). GM1-Pentasaccharide was obtained from Carbosynth (Compton, UK). An antagonist for chemokine receptor CCR2 (RS 102895) was purchased from Santa Cruz Biotechnology Inc. (Santa Cruz, CA, USA). *D*-(+)-Mannose, *N*-Acetyl-D-galactosamine, *D*-(+)-Galactose and *D*-(+)-Glucose were obtained from Sigma-Aldrich (St. Louis, MO, USA). Matrigel was purchased from BD Biosciences (Franklin Lakes, NJ, USA). Heparin was obtained from JW Pharmaceutical (Seoul, Korea). Antibodies were purchased as follows: Anti-Arginase (Arg) 1, mannose receptor (CD206), YM1, MCP-1, IL-10, interferon gamma (IFN-γ), IL-2 receptor gamma (γC), and integrin αV were purchased from Abcam (Cambridge, UK). Anti-p-STAT3, p-STAT5, TNF-α, and IL-1β were obtained from Cell Signaling Technology (Danvers, MA, USA). Anti-p-STAT1, p-STAT6, STAT, STAT3, STAT5, STAT6, F4/80, VEGF, and GAPDH were purchased from Santa Cruz Biotechnology Inc. Anti-β-actin was obtained from Sigma-Aldrich. Anti- nitric oxide synthase (iNOS) was purchased from Millipore (CA, USA).

### Cell culture

Lewis lung carcinoma cells (LLC), melanoma cells (B16-F10) and Raw264.7 cells (American Type Culture Collection, Manassas, VA, USA) were cultured in Dulbecco's modified Eagles medium/high glucose (DMEM; GE Healthcare Life Sciences, Buckinghamshire, UK.) containing 10% heat-inactivated fetal bovine serum (FBS; Sigma-Aldrich), 100 U/mL penicillin, and 100 μg/mL streptomycin (Invitrogen^™^ Thermo Fisher Scientific, Waltham, MA, USA) and maintained in a humidified incubator at 37°C and 5% CO_2_ prior to the experiment. Colon carcinoma cells (CT26; American Type Culture Collection) were cultured in RPMI 1640 Medium (GE Healthcare Life Sciences). Murine peritoneal macrophages were isolated as previously described [53]. Brewer thioglycollate medium (1 mL of 3%; BD Biosciences) was injected into the peritoneal cavity of the mice. After 4 days, the mice were euthanized by CO_2_ inhalation, and 10 mL cold 1 × PBS were injected into each mouse for peritoneal cavity lavage. The peritoneal fluids were collected and centrifuged at 400 × *g* for 10 min. The cell pellets were washed and resuspended with DMEM containing 10% FBS, 100 U/mL penicillin and 0.1 mg/mL streptomycin. The cells were cultured for the experiments. Murine BMDM were prepared as previously described [54, 55]. Briefly, after euthanizing the mice with CO_2_, cellular material from the femurs was collected, and pressed through a nylon mesh filter (30 μm; Merck Millipore, Billerica, MA, USA), and centrifuged at 400 × *g* at 4°C for 5 min. Next, the cells were re-suspended in RPMI1640 containing 10% FBS, 100 U/mL penicillin, 0.1 mg/mL streptomycin, and 50 ng/mL macrophage-colony stimulating factor (M-CSF; PeproTech Inc., NJ, USA). After incubation at 37°C and 5% CO_2_ for 24 h, the cells were washed 3 times with RPMI1640 to remove non-adherent cells and cultured for 1 week: RPMI1640 was subsequently replaced every 2 days. The cells were then detached, washed, counted, and cultured for the experiments. HUVECs, obtained from Cambrex Bio Science (East Rutherford, NJ, USA), were cultured in sterile endothelial cell growth medium (EGM-2, Cambrex Bio Science) and were maintained as described previously [29].

### Reverse-transcription polymerase chain reaction (RT-PCR)

The total RNA of the cells was isolated with a GeneJET RNA Purification Kit (Thermo Scientific). One microgram of total RNA was reverse-transcribed using RevertAid reverse transcriptase (Thermo Fisher Scientific), and single-stranded cDNA was amplified by PCR using AccuPower^®^ PCR PreMix (Bioneer, Daejeon, Korea). The PCR-amplified size of each target gene, and the primers used in this study were shown in Table S1. The PCR products were separated by electrophoresis on 1.5% agarose gel containing ethidium bromide with 1 × Tris-acetate buffer and visualized under UV light.

### Western blot analysis

Membrane proteins and cytosolic proteins were isolated using hypotonic buffer containing 20 mM HEPES (pH 7.4), 5 mM KCl, 1 mM MgCl_2_, 1 mM DTT and 1% NP-40 cell lysis buffer [150 mM NaCl, 10 mM HEPES (pH 7.4), 1% NP-40, 5 mM NaPyrophosphate, 5 mM NaF, 2 mM NaOrthovanadate]. Total proteins were isolated using 1% NP-40 cell lysis buffer containing a protease inhibitor cocktail tablet (Roche Applied Science, Penzberg, Germany) and the protein content was measured by Bradford′s method (Bio-Rad Protein Assay; Bio-Rad Laboratories, Hercules, CA, USA). Equal amounts of protein from each sample were electrophoresed by SDS-PAGE, and transferred to nitrocellulose membranes (GE Healthcare Life Sciences). The membranes were blocked for 1 h with 5% nonfat dry milk prior to incubation with target protein-specific primary antibodies at 4°C overnight. After incubation with secondary antibodies conjugated with horseradish peroxidase at room temperature for 1 h, the bands of interest were revealed using a luminescent image analyzer (ImageQuant LAS 4000; GE Healthcare Life Sciences).

### Arginase activity assay

Arginase activity in the cell lysates was determined using a QuantiChrom^™^ Arginase Assay Kit (DARG-200; BioAssay Systems, Hayward, CA, USA). Briefly, cells pellets (1 × 10^6^ cells/6 well) were lysed with 10 mM Tris buffer (pH 7.4) containing 1 μM pepstatin A, 1 μM leupeptin, 0.4% (w/v) Triton X-100. These mixtures were centrifuged for 10 min at 14,000 × *g*, and the supernatants were used for arginase activity. Forty microliters of each sample were added to 10 μL of arginase substrate buffer, and incubated at 37°C for 2 h. The reaction mixtures were incubated with 200 μL urea reagent for 60 min at room temperature, and optical density at 430 nm was determined using a SpectraMax M2 microplate reader (Molecular Devices, Sunnyvale, CA, USA). The manufacturer's urea standard (50 mg/dL) was used to calculate arginase activity.

### *CD206* knockdown by shRNA

To knock down endogenous mouse CD206, mCD206 shRNA constructs (5 clones) were obtained from Open Biosystems (Thermo Scientific). Raw264.7 cells (1 × 10^6^) were subcultured on a 6-well plate. Twenty-four hours after cell seeding, mCD206 shRNA (3 μg) was transfected into Raw264.7 cells using Lipofectamine 2000 (Invitrogen). Twenty-four hours after transfection, cells with stably integrated mCD206 shRNA were selected with 3 μg/mL puromycin treatment for 1 week. The knockdown efficiency of the mCD206 shRNA was verified by western blot analysis. The best performing mCD206 shRNA #5, among 5 mCD206 shRNA clones (#1–#5), was used for subsequent experiments. The sequence of mCD206 shRNA #5 was 5′-AAGATCCAGATAAACACATGC-3′.

### The isolation of membrane proteins and GM1-biotin/avidin pull down assay

To isolate crude membrane proteins, Raw264.7 cells (1.5 × 10^8^ cells) were suspended in ice-cold hypotonic buffer. The cells were homogenized using a 26G needle and 1 mL syringe, and centrifuged at 20,000 × *g* for 10 min at 4°C. The pellets were collected, suspended in 1 mL 1% NP-40 cell lysis buffer, and centrifuged at 20,000 × *g* for 10 min at 4°C. The supernatant was divided into two aliquots of 500 μL. GM1-biotin or biotin (20 μM) was added to the crude membrane proteins and incubated at 4°C overnight a on rotary-shaking machine, followed by incubation with 30 μL Neutravidin^®^ Agarose Resin (Thermo Fisher Scientific) for 2 h at 4°C. Protein-resin complexes were washed five times with 1 % NP-40 cell lysis buffer and released from the beads by boiling in a 6 × SDS sample buffer [125 mM Tris–HCl (pH 6.8), 4% SDS, 10% β-mercaptoethanol, 2% glycerol, and 0.02% bromophenolblue] for 5 min. The reaction mixture was resolved on an 8% SDS–PAGE gel, transferred onto a nitrocellulose membrane by electroblotting, and then probed with anti-CD206, anti-γC, and integrin αV antibodies.

### Tube formation assay

To investigate the formation of a capillary-like network of HUVECs by factors secreted from macrophages treated with GM1, the tube formation assay was performed as we previously described study [29] with some modifications, using 24-well chambers containing polycarbonate filter inserts (Corning Inc., Corning, NY, USA). Matrigel (13.9 mg/mL) was thawed at 4°C, and mixed with EBM-2 medium at a 1:1 ratio. The 70 μL of EBM-2-diluted Matrigel (6.95 mg/mL) was added to each well of the 24-well culture plates, and allowed to polymerize at 37°C for 1 h. The HUVECs, to be tested for tube formation, were detached from tissue culture plates, washed, resuspended in DMEM/EBM-2 medium (1:1) containing 1% FBS (1 × 10^4^ cells/well), and seeded into the Matrigel-coated wells. Raw264.7 cells (1 × 10^6^ cells) suspended in DMEM were seeded on the upper sides of the filters with polycarbonate filter inserts, and treated with or without GM1. After incubation for 12 h at 37°C in a 5% CO_2_ atmosphere, capillary-like tube formation in each well of the culture plates was photographed with a Nikon light microscope.

### Matrigel plug assay

The Matrigel plug assay was performed as described previously [22, 29]. C57BL/6 mice were subcutaneously injected with 500 μL of a Matrigel (400 μL) and heparin (10 Unit/mL) mixture with Raw264.7 cells (1 × 10^7^ cells/100 μL PBS) in the presence or absence of GM1 (20 μM). After 7 days, the mice were euthanized, and the Matrigel plugs were removed, fixed with 3.7% formalin in PBS, embedded in paraffin, and then cut into 4-μm serial sections. The sections were stained with hematoxylin and eosin (H&E) solution for microscopic observation.

### Animals

Male C57BL/6 mice, inbred in a specific pathogen-free facility, were purchased from Orient Bio (Seongnam, Korea). The animals were housed in certified, standard laboratory cages, and fed with food and water *ad libitum* prior to the experiment. All experimental procedures followed the Guidelines for the Care and Use of Laboratory Animals of the National Institutes of Health of Korea, and all experiments were approved by the Institutional Animal Care and Use Committee of Pusan National University, Pusan, Republic of Korea.

### Statistical analysis

The values for the arginase activity and cytokine assay were calculated as a percentage of the control cell values and expressed as mean ± SD. The differences between the mean values and control groups were evaluated by student's *t*-test and analysis of variance with an unpaired *t* test. The minimum level of significance was set at a *p* value of 0.05 for all analyses. All experiments were carried out at least 3 times, independently.

## SUPPLEMENTARY FIGURES AND TABLE


