# Supplementary material for: The function of cancer-shed gangliosides in macrophage phenotype: involvement with angiogenesis
Source: Oncotarget. 2016 Dec 10;8(3):4436–48. doi: 10.18632/oncotarget.13878 (PMC5354844; doi:10.18632/oncotarget.13878)
Supplement: Supplementary file 1 [file oncotarget-08-4436-s001.pdf]

The function of cancer-shed gangliosides in macrophage phenotype: involvement with angiogenesis

Supplementary Materials

MATERIALS AND METHODS

Measurement of cytokines, chemokines, and growth factors

The conditioned culture medium from Raw264.7 cells treated with or without GM1, was collected, and stored at −80°C before use for the cytokine measurement. The 14 analytes including the granulocyte-macrophage colony-stimulating factor (GM-CSF), interferon (IFN)-γ, IL-10, IL-1α, IL-1β, IL-4, IL-6, keratinocyte-derived chemokine (KC), MCP-1, macrophage colony-stimulating factor (M-SCF), regulated on activation normal T cell expressed and secreted (RANTES), TNF-α, vascular endothelial growth factor (VEGF), and IL-13, were measured using the commercially available Luminex multiplexing system (Koma Biotech, Seoul, Korea). The levels of each cytokine evaluated in the samples were expressed as pg/mg.

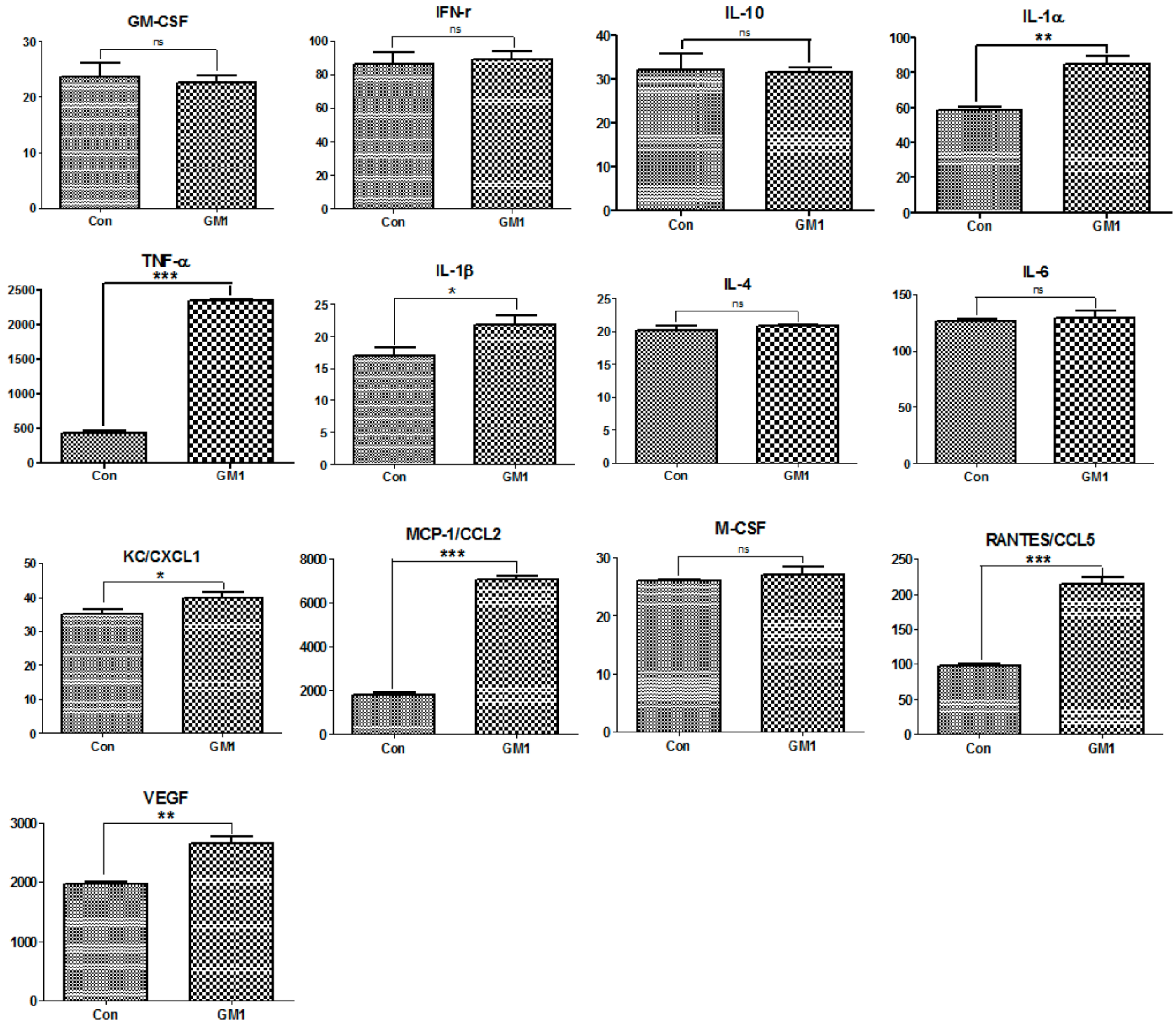

Supplementary Figure S1: The effects of GM1 on production of cytokines and growth factors in Raw264.7 cells. Raw264.7 cells were treated with GM1 (10 nM) for 24 h, the media were collected, and subjected to Luminex multiplex assay for measuring production of multiple cytokines and growth factors.

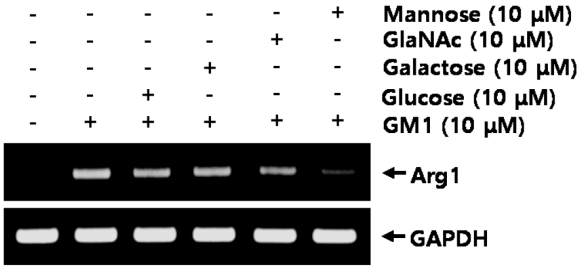

Supplementary Figure S2: Inhibition of GM1-stimulated arginase-1 expression by treatment of diverse monosaccharides. Raw264.7 cells were treated with each monosaccharides (10 μM) for 1 h prior to GM1 (10 μM) treatment. After 24 h, the cells were harvested, and the expression of arginase-1 was estimated by RT-PCR analysis.

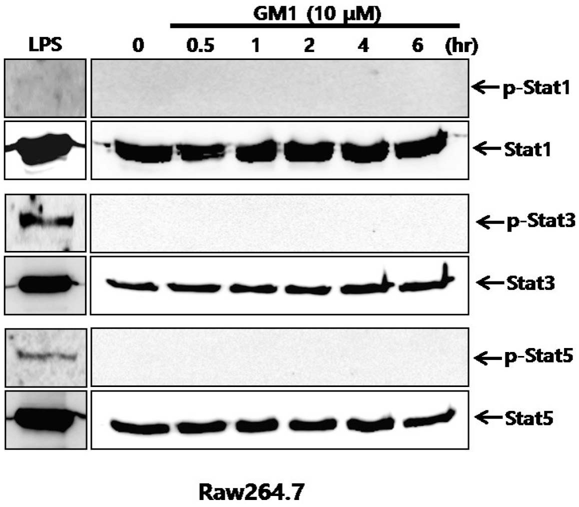

Supplementary Figure S3: GM1 treatment does not affect the phosphorylation of STAT1, 3, and 5. Raw264.7 cells were treated with LPS or GM1 (10 μM) for indicated times. The phsphorylation of STAT1, 2, 5 was measured by Western blot analysis.

Supplemenytary Table S1: Amplified size for each target gene, and the primers used in this study

Gene	Primer Sequences	Size (bp)
Arg-1	Forward: 5′-ACCTGCTGGGAAGGAAGAAA-3′<br>Reverse: 5′-ATGGTTACCCTCCCGTTGAG-3′	284 bp
YM1	Forward: 5′-ACTGGAATTGGTGCCCCTAC-3′<br>Reverse: 5′-TTGTCCTTGAGCCACTGAGC-3′	221 bp
F4/80	Forward: 5′-TCGTGCTGGAGCAAGCGACC-3′<br>Reverse: 5′-CCCGGTCACAGTGCCACCAA-3′	404 bp
CD206	Forward: 5′-GCGCTGCGTGGACGCTCTAA-3′<br>Reverse: 5′-CCAGAGCCATCCGTCCGAGC-3′	481 bp
VEGF	Forward: 5′-CAGCACATAGGAGAGATGAGC-3′<br>Reverse: 5′-TCACCGCCTCGGCTTGTCACA-3′	234, 306 bp
IL-10	Forward: 5′-ACCTGGTAGAAGTGATGCCCCAGGCA-3′<br>Reverse: 5′-CTATGCAGTTGATGAAGATGTCAAA-3′	237 bp
TNF-α	Forward: 5′-GGCAGGTCTACTTTGGAGTCATTGC-3′<br>Reverse: 5′-ACATTCGAGGCTCCAGTGAATTCGG-3′	307 bp
IL-1β	Forward: 5′-GCCCATCCTCTGTGACTCAT-3′<br>Reverse: 5′-AGGCCACAGGTATTTTGTCG-3′	230 bp
iNOS	Forward: 5′-CTGCAGCACTTGGATCAGGAACC-3′<br>Reverse: 5′-GGGAGTAGCCTGTGTGCACCTGGAA-3′	311 bp
IFN-γ	Forward: 5′-TCTGGGCTTCTCCTCCTGCGG-3′<br>Reverse: 5′-GGCGCTGGACCTGTGGGTTG-3′	421 bp
GAPDH	Forward: 5′-AACTTTGGCATTGTGGAAGG-3′<br>Reverse: 5′-ACACATTGGGGGTAGGAACA-3′	223 bp
